# Spices for Prevention and Treatment of Cancers

**DOI:** 10.3390/nu8080495

**Published:** 2016-08-12

**Authors:** Jie Zheng, Yue Zhou, Ya Li, Dong-Ping Xu, Sha Li, Hua-Bin Li

**Affiliations:** 1 Guangdong Provincial Key Laboratory of Food, Nutrition and Health, School of Public Health, Sun Yat-Sen University, Guangzhou 510080, China; zhengj37@mail2.sysu.edu.cn (J.Z.); zhouyue3@mail2.sysu.edu.cn (Y.Z.); liya28@mail2.sysu.edu.cn (Y.L.); xudp@mail2.sysu.edu.cn (D.-P.X.); 2 School of Chinese Medicine, The University of Hong Kong, Hong Kong, China; u3003781@connect.hku.hk; 3 South China Sea Bioresource Exploitation and Utilization Collaborative Innovation Center, Sun Yat-Sen University, Guangzhou 510006, China

**Keywords:** spice, cancer, curcumin, thymoquinone, capsaicin

## Abstract

Spices have been widely used as food flavorings and folk medicines for thousands of years. Numerous studies have documented the antioxidant, anti-inflammatory and immunomodulatory effects of spices, which might be related to prevention and treatment of several cancers, including lung, liver, breast, stomach, colorectum, cervix, and prostate cancers. Several spices are potential sources for prevention and treatment of cancers, such as *Curcuma longa* (tumeric), *Nigella sativa* (black cumin), *Zingiber officinale* (ginger), *Allium sativum* (garlic), *Crocus sativus* (saffron), *Piper nigrum* (black pepper) and *Capsicum annum* (chili pepper), which contained several important bioactive compounds, such as curcumin, thymoquinone, piperine and capsaicin. The main mechanisms of action include inducing apoptosis, inhibiting proliferation, migration and invasion of tumors, and sensitizing tumors to radiotherapy and chemotherapy. This review summarized recent studies on some spices for prevention and treatment of cancers, and special attention was paid to bioactive components and mechanisms of action.

## 1. Introduction

Spices have been widely used as condiments for thousands of years because of their flavor, taste and color. Several spices have been used as medicinal plants in folk medicine for the treatment of various diseases because they contain many bioactive compounds and possess a lot of beneficial health effects. For example, some antioxidants from spices, such as curcumin (turmeric), eugenol (clove), and capsaicin (red pepper), were experimentally evidenced to control cellular oxidative stress due to their antioxidant properties and their capacity to block the production of reactive oxygen species and interfering with signal transduction pathways [1,2]. Besides, inflammatory processes were modulated by spice compounds such as curcumin and thymoquinone [3,4]. In addition, spices were sometimes used as a source of alternative antimicrobial strategies, including some spices belonging to the genus Cinnamomum [5]. Moreover, the immunomodulatory effects of some spice compounds were confirmed, such as thymoquinone [4]. In a word, the antioxidant, anti-inflammatory, and immunomodulatory effects of spices have been confirmed in many studies [6]. Therefore, spices could be used to prevent and treat cancers, because oxidative stress [7,8,9,10,11,12], inflammatory stress [13,14] and immune response [15,16] have been associated with the genesis, growth, and metastasis of cancers [17,18,19]. In fact, epidemiological and experimental evidences have shown that certain spices might lower risks of some cancers [20,21,22,23].

Cancer is one of the major causes of death in the world, with approximately 14 million new cases and 8.2 million cancer-related deaths worldwide in 2012 [24], and the number of new cases is expected to rise by about 70% over the next two decades. Surgery, radiotherapy and chemotherapy are the major treatment modalities. However, surgery alone, or radiation alone, is effective only when the tumor is localized and small in size, and chemotherapy alone could be effective for a small sized tumor. Besides, they might induce certain side effects. So, there is a real need for new anticancer drugs with reduced side effects, and spices are a promising source and could treat chemotherapy-induced indigestion, nausea, vomiting, and metallic taste [25,26].

This review summarized some studies about spices-derived substances exhibiting anti-cancer and chemoprevention activity, and their mechanisms of action. Also, some suggestions and prospects for future studies have been offered.

## 2. Turmeric and Curcumin

**Figure 1:**
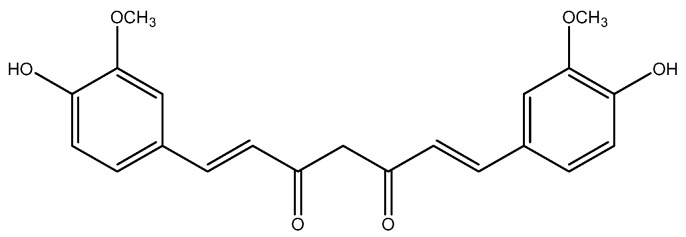
Structure of curcumin.

Turmeric (*Curcuma longa*) is used as a spice and gives a specific flavor and yellow color in Asian food. Curcumin (Figure 1), a polyphenolic compound, is a secondary metabolite isolated from the rhizomes of turmeric, and exhibits a number of therapeutic effects, including anti-cancer properties, via modulating different molecular regulators [27,28,29].

### 2.1. Nasopharyngeal Cancer

Curcumin induced G_2_/M phase arrest and apoptosis in human nasopharyngeal carcinoma cells (NPC), which have been associated with mitochondria, apoptosis inducing factor and caspase-3-dependent pathways [30]. Besides, the radiosensitivity of NPC was induced by curcumin, which was associated with long non-coding RNAs (lncRNAs) profiles. Expression of lncRNA and mRNA was significantly reversed by curcumin. Curcumin enhanced radiosensitization by reversing irradiation (IR)-induced differentially expressed lncRNAs in NPC cells, which suggested that lncRNAs had important functions in IR-induced radioresistance [31]. In another study, curcumin exhibited inhibitory effects on NPC via inhibiting the expression of miR-125a-5p and enhancing the expression of tumor protein 53 (TP53) gene [32].

In a translational study, curcumin treatment could inhibit proliferation of NPC through altering expression of proteins in the extracellular regulated protein kinase (ERK)-1/2 signaling pathway in mouse xenografts [33].

### 2.2. Lung Cancer

In a study, Bax expression was increased while the expression of B-cell lymphoma-2 (Bcl-2) and B-cell lymphoma-xL (Bcl-xL) was decreased by curcumin in small cell lung cancer, thus inducing apoptosis accompanied by increasing intracellular reactive oxygen species (ROS) levels. Mitochondrial membrane potential was decreased, the release of cytochrome c into the cytosol was induced, and then caspase-9 and caspase-3 were activated [34]. Besides, curcumin inhibited the enzymatic activity of the epidermal growth factor receptor (EGFR) intracellular domain, and also influenced the cell membrane environment of EGFR, which was involved in the growth of lung cancer [35]. According to another study, the crosstalk between adherens’ junctions and Wnt signaling pathways was regulated by early growth response 1 (EGR-1), suggesting that EGR-1 might regulate cell proliferation and migration, which was confirmed in vitro. At the same time, the curcumin-induced decrease of EGR-1 was validated, which was involved in the anti-proliferation and anti-migration activity of curcumin in non-small cell lung cancer (NSCLC) cells [36]. According to Ye et al., the signal transducers and activators of transcription 3 (STAT3) was a therapeutic target in some squamous cell lung carcinoma (SCC) tumors, and protein inhibitor of activated STAT3 (PIAS3) was an endogenous inhibitor of STAT3, which was inhibited in SCC tumors cell lines. Endogenous PIAS3 expression was increased by curcumin treatment, and cell growth and viability were also decreased in Calu-1 cells, a model of SCC [37]. Additionally, curcumin-induced apoptosis was associated with miR-192-5p/215 induction; X-linked inhibitor of apoptosis (XIAP) was a transcriptional target of miR-192-5p/215, indicating p53-miR-192-5p/215-XIAP pathway was an important therapeutic target of curcumin for NSCLC [38]. In another study, it was validated that curcumin treatment to human lung cancer cells could induce DNA damage and inhibit expression of DNA-repair-associated proteins, such as breast cancer susceptibility gene 1 (BRCA1), 14-3-3 protein σ, O6-methylguanine-DNA methyltransferase (MGMT), and mediator of DNA damage checkpoint 1 (MDC1) [39].

Some translational studies on animals also confirmed the anti-tumor effects of curcumin in lung cancer. For example, curcumin remarkably inhibited tumor growth of orthotopic human NSCLC xenografts and increased survival of treated athymic mice, and significantly decreased survival and increased induction of apoptosis in NSCLC cells through inhibiting COX-2, p65 expression and ERK1/2 activity [40,41]. In addition, curcumin could inhibit JAK2 activity and reduce tumor spheres via inhibiting the JAK2/STAT3 signaling pathway. Thus, curcumin strongly repressed tumor growth in the lung cancer xenograft nude mouse model [42].

### 2.3. Hepatobiliary Cancer

Hypoxia-inducible factor (HIF)-1 is a transcription factor playing a central part in cell survival and angiogenesis in hypoxic tumors, composed of HIF-1α and aryl hydrocarbon receptor nuclear translocator (ARNT/HIF-1β). HIF-1α and HIF-2α protein levels were lowered by curcumin in hypoxia, and ARNT protein levels and HIF transcriptional activity were reduced in normoxia and hypoxia by curcumin, and survival of Hep3B hepatoma cells was negatively impacted [43]. Diethylnitrosamine (DENA)-induced hepatocarcinogenesis and damage were prevented and reversed by curcumin in rats [44,45]. Curcumin remarkably suppressed the serum levels of α feto-protein (AFP), interleukin-2 (IL-2), interleukine-6 (IL-6), alanine amino transferase (ALT), and malondialdehyde (MDA) as well as gene expression of IL-2 and IL-6, and increased the gene expression and activities of glutathione peroxidase (Gpx), glutathione reductase (GR), catalase (CAT) and super oxide dismutase (SOD) [44]. Moreover, the overexpression of the angiogenic and anti-apoptotic factors transforming growth factor-β (TGF-β) and protein kinase B (PKB) were reduced, while caspase-3 expression was improved. Liver marker enzymes aspartate aminotransferase (AST) and ALT and lipid peroxidation were normalized [45]. In addition, curcumin inhibited the growth of liver cancer in a dose-dependent manner in nude mice [46].

Attention was also paid to cholangiocarcinoma. Curcumin induced antiproliferation and apoptosis in cholangiocarcinoma cells. The apoptosis was significantly related to production of superoxide anion, while the up-regulation of tumor protein 53 (P53) and Bcl-2 associated X protein (Bax) were associated with oxidative stress and apoptosis [47]. Besides, curcumin-treated cholangiocarcinoma cells exhibited reduced viability compared with control treatment. Expression of cleaved poly (ADP) ribose polymerase and caspase activity was increased, showing that apoptosis was induced by curcumin [48].

### 2.4. Breast Cancer

According to Strofer et al., HIF-1α and HIF-2α protein levels in hypoxia were lowered by curcumin. Curcumin also reduced ARNT protein levels and HIF transcriptional activity both in normoxia and hypoxia in MCF-7 breast carcinoma cells [43]. Additionally, 12-O-tetradecanoylphorbol-13-acetate (TPA)-induced matrix metalloproteinase (MMP)-9 expression and cell invasion were inhibited by curcumin through the suppression of protein kinase C-α (PKC-α), mitogen-activated protein kinase (MAPK) and nuclear factor-κB/active protein-1 (NF-κB/AP-1) pathway [49]. Besides, breast cancer stem cells could aggravate migration due to the suppression of E-cadherin, which was restored by curcumin through inhibiting β-catenin nuclear translocation [50]. Triple negative breast cancer is an aggressive breast cancer phenotype with a poor prognosis. It lacks expression of the estrogen receptor, progesterone receptor and epidermal growth factor receptor 2 (EGFR2). Curcumin was able to inhibit the proliferation of triple negative breast cancer cells, probably through inhibiting the EGFR signaling pathway [51,52]. Additionally, the combination of curcumin and 5-fluorouracil (5-FU) protected normal cells from reduced viability and permitted higher dosing or longer treatment times of 5-FU, thus augmenting the chemotherapeutic effectiveness of 5-FU which was an antimetabolite with cytotoxic side effects [53]. In addition, retinoic acid resistant triple negative breast cancer cells to retinoic acid were also sensitized by curcumin [54].

The anti-tumor effects of curcumin in breast cancer were studied in animal models. Curcumin-induced inhibition of tumor growth and angiogenesis in mouse model was related to down-regulating the expression of cyclin D1, platelet endothelial cell adhesion molecule-1 (PECAM-1), and p65 [55]. Another animal study showed curcumin suppressed metastatic breast cancer in mice by changing m1/m2 macrophage balance in the tumor micro-environment [56]. In addition, curcumin treatment led to a decrease in tumor volume and cell proliferation in the xenograft model of breast cancer [55,57].

Several clinical studies researched the effects of curcumin in the breast cancer. In a randomized, double-blind, placebo-controlled clinical trial, oral curcumin (6.0 g/day) during radiotherapy was found to reduce the severity of radiation dermatitis in breast cancer patients [58]. Another study showed that curcumin could be used as an in vivo inhibitor of breast cancer resistance protein [59]. In addition, the recommended dose of curcumin is 6000 mg/day for seven consecutive days every 3 weeks in combination with a standard dose of docetaxel for the combination therapy in advanced and metastatic breast cancer patients [60].

### 2.5. Gastric Cancer

Curcumin induced loss of mitochondrial membrane potential and increased the cell apoptotic rate in gastric cancer cells, which was related with impaired ATP-sensitive potassium channel (KATP) opening [61]. Besides, the combination of Kruppel-like factor 4 (KLF4) overexpression and curcumin had significant anti-proliferation, pro-apoptosis and anti-invasion effects on human gastric carcinoma cells, indicating that KLF4 was a potential therapeutic target, and curcumin was a promising therapeutic drug in stomach cancer [62]. In addition, lymphatic vessel density was reduced by curcumin in an in vivo human gastric cancer model, lymphatic vessel endothelial receptor 1 (LYVE-1), prospero homeobox 1 (Prox-1), podoplanin, and vascular endothelial growth factor receptor 3 (VEGFR-3) mRNA expression were down-regulated, which indicated gastric cancer lymph node metastasis might be inhibited by curcumin [63]. Besides, curcumin inhibited cancer cell growth, induced cell cycle arrest at G_2_/M phase, and down-regulated glycolytic enzymes expressions, thus blocking cell growth [64]. Additionally, an in vivo study showed that the combination of curcumin and 5-FU/oxaliplatin exhibited potent growth inhibition of BGC-823 xenograft tumors [65].

### 2.6. Colorectal Cancer

Curcumin prevented aberrant crypt foci (ACF) and adenomas in murine models of colorectal carcinogenesis, which involved inhibiting production of mucosal concentrations of pro-carcinogenic eicosanoids 5-hydroxyeicosatetraenoic acid (5-HETE) and prostaglandin E-2 (PGE-2) [66], and curcumin-induced apoptosis could be reversed by PGE-2 in colon cancer cells [67]. In addition, curcumin inhibited the growth of human colon adenocarcinoma cell lines and induced apoptosis as evidenced by nuclear fragmentation as well as condensation and DNA fragmentation [68]. According to another research, the expression and activity of hexokinase II were down-regulated by curcumin, and curcumin induced-dissociation of hexokinase II from the mitochondria led to mitochondrial-mediated apoptosis [69]. Moreover, the epigenetic demethylation and up-regulation of deleted in lung and esophageal cancer 1 (DLEC1), a tumor suppressor gene, could be involved in the inhibitory effect of curcumin on anchorage-independent growth of human colon cancer cells [70]. Additionally, the chemoresistance of colorectal cancer to 5-FU resulted from epithelial-mesenchymal transition (EMT), and combination of curcumin and 5-FU enhanced cellular apoptosis and inhibited proliferation in 5-FU resistant cells. Curcumin treatment up-regulated EMT-suppressive miRNAs in 5-FU resistant cells [71].

Several clinical studies researched the effects of curcumin on breast cancer. For instance, in a phase IIa clinical trial of curcumin for the prevention of colorectal neoplasia, curcumin was well tolerated at both 2 g and 4 g in patients, and could decrease ACF number [66]. In addition, curcumin treatment improved the general health of patients with colorectal cancer via up-regulating p53 molecule expression in tumor cells and consequently speeded up tumor cell apoptosis [72].

### 2.7. Prostate Cancer 

Resurgent activity of androgen receptor (AR) and the up-regulation of coactivator protein p300 and cAMP response element-binding protein (CBP) resulted in aggressive phenotypes and hormone therapy failures in prostate cancer. Curcumin suppressed CBP and p300 occupancy at sites of AR function through reducing histone acetylation and altering the chromatin landscape, thus reducing tumor growth and delaying the onset of castrate-resistant disease [73]. In addition, the proliferation of prostate cancer cells (PC-3) and growth of xenografted tumors (mouse model) were inhibited through down-regulation of inhibitor of DNA binding 1 by small interfering RNA [74]. Moreover, severe combined immune deficiency (SCID) mice with PC-3 xenograft tumors were treated with α-tomatine and curcumin. The growth of PC-3 tumors was more potently inhibited than either agent alone [75].

### 2.8. Cancer in Uterus

After treatment with curcumin-based cervical cream, HPV^+^ cervical cancer cells were selectively eliminated, antigen E6 transformation and EGFR expressions were inhibited, and concomitantly p53 was induced. It showed that curcumin-based vaginal cream on the vaginal epithelium of healthy mice eradicated HPV^+^ cancer cells and did not affect non-cancerous tissue [76]. Additionally, the proliferation and apoptosis of human endometrial carcinoma cells was down-regulated by curcumin through down-regulating their AR expression mediated by the Wnt signal pathway [77]. In another study, curcumin induced decrease in silver-staining nucleolar organizer region (AgNOR) protein pools which reflected the rapidity of cancer cell proliferation, which might be mediated by global DNA hypermethylation in HeLa cells [78]. Besides, tumor growth and angiogenesis in cervical cancer-implanted mice were inhibited by curcumin via down-regulating vascular endothelial growth factor (VEGF), cyclooxygenase-2 (COX-2) and EGFR [79].

### 2.9. Hematopoietic Tumor

Curcumin, or in combination with other drugs, increased cell death in hematopoietic tumor cells [27]. For example, the Wilms’ tumor 1 (WT1) gene is a regulating factor in cell proliferation, which is highly expressed in patients with acute myeloid leukemia [80,81]. WT1 (+/+) mRNA level was strongly inhibited by curcumin, and exogenous WT1 (+/+) protein half-life was also decreased, via protein kinase C during post-translational processing [80]. In another study, cell proliferation and clonogenicity were suppressed, and cell cycle arrest at the G_2_/M phase was induced by curcumin with reduction in the WT1 levels [81]. Moreover, in CCRF-CEM human T-cell leukemia cells (lymphatic), DNA plasmids were mostly damaged after treatment with curcumin in the presence of Cu^2+^, while curcumin or Cu^2+^ alone failed to cause DNA damage [82]. In addition, curcumin could inhibit the growth and promote apoptosis of leukemic cells which were derived from acute promyelocytic leukemia. The apoptosis induced by curcumin was through an amplification of endoplasmic reticulum (ER) stress, which possibly resulted from the accumulation of misfolded nuclear receptor corepressor protein in the ER [83]. In another study, in Burkitt’s lymphoma cell lines, ionizing radiation-induced apoptosis and G_2_/M phase arrest were increased by pretreatment of curcumin through NF-κB pathway [84]. Additionally, in HuT-78 cells (T-cell lymphoma), curcumin-mediated rapid generation of ROS induced apoptosis through modulating different cell survival and cell death pathways [85].

### 2.10. Other Cancers

The effects of curcumin on other cancers were also studied. For instance, proliferation and migration were inhibited and cell death was induced by curcumin in models of glioblastoma. The constitutive activation of phosphatidylinositol 3-kinase (PI3K)/PKA and NF-κB survival pathways were decreased by curcumin, accompanied by down-regulating the antiapoptotic NF-κB-regulated protein Bcl-xl and inducing mitochondrial dysfunction as a prelude to apoptosis [86]. In addition, cell growth, migration and invasion in pancreatic cancer were suppressed by curcumin, and cell apoptosis was induced, which is associated with increased expression of miR-7 and subsequently decreased expression of SET8, one of the miR-7 targets [87]. Besides, the viability, cell attachment, spreading, migration and invasion abilities of K1 papillary thyroid cancer cells were suppressed, hypoxia-induced ROS up-regulation was inhibited by curcumin, and the mRNA and protein expression levels of HIF-1α were decreased in K1 cells. E-cadherin expression was enhanced, and MMP-9 enzyme activity was inhibited [88,89]. Moreover, curcumin up-regulated pro-apoptotic Bik, down-regulated survival signaling by Akt and NF-κB in head and neck squamous cell carcinoma cell lines [90]. Curcumin plays an important role in treatment of many other cancers, such as, peripheral nerve sheath tumors [91], and oral squamous cell carcinoma [92].

## 3. *Nigella sativa* and Thymoquinone

**Figure 2:**
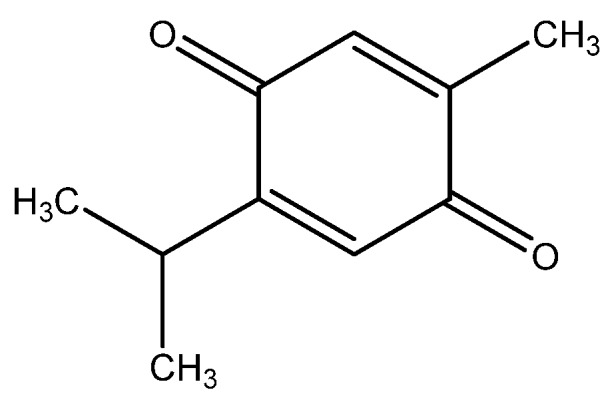
Structure of thymoquinone.

*Nigella sativa* L., commonly referred as black cumin, is an oriental spice that has been used since the times of ancient Egypt. It is an annual herb growing in countries bordering the Mediterranean Sea and India, and is used as a natural medicine for treatment of many acute as well as chronic conditions ranging from fever to intestinal disturbances to cancer [93,94,95,96]. Thymoquinone (Figure 2) is the predominant bioactive constituent isolated from black seeds of *Nigella sativa* and has been shown to possess antineoplastic activity against multifarious tumors [97]. 

### 3.1. Lung Cancer

The seed extract and seed oil of *Nigella sativa* were found to significantly reduce the cell viability and altered the cellular morphology of human lung cancer cells in a concentration dependent manner [98]. In addition, thymoquinone played a role in inhibiting the proliferation, migration, and invasion of A549 lung cancer cells, and the expression of proliferating cell nuclear antigen, cyclin D1, MMP-2, and MMP-9 was inhibited by thymoquinone through ERK-1/2 pathway [99]. Moreover, in a mouse xenograft model, a combination of thymoquinone and cisplatin was well tolerated and remarkably reduced tumor volume and tumor weight without additional toxicity to the mice [100].

### 3.2. Hepatobiliary Cancer

Thymoquinone had a potent anti-proliferative activity by regulating the G_1_/S phase cell cycle transition and exhibited a beneficial role in the treatment of hepatocellular carcinogenesis [101,102]. Moreover, thymoquinone inhibited the growth of human cholangiocarcinoma cell lines, induced cell cycle arrest, and promoted apoptosis. The thymoquinone-induced anticancer effect was due to down-regulation of PI3K/Akt and NF-κB regulated gene products, including p-Akt, p65, XIAP, Bcl-2, COX-2, and VEGF [102].

### 3.3. Breast Cancer

The anti-proliferative and pro-apoptotic effects of thymoquinone were associated with inducing p38 phosphorylation via ROS production [103] and inhibiting Akt kinases which were usually hyper-activated in tumor cells [104]. In addition, combined with tamoxifen, thymoquinone led to a substantial increased apoptosis and marked inhibition of cell growth in breast cancer, which resulted in regulation of multiple cell signaling targets including inactivation of Akt and degradation of XIAP, an endogenous inhibitor of apoptosis by inactivating key caspases [105]. Moreover, the growth inhibitory effects of thymoquinone on triple negative breast cancer cell lines with mutant p53 involved reduction of Akt phosphorylation and decreased expression of XIAP. Cisplatin- and docetaxel-induced cytotoxicity was also augmented by thymoquinone [106]. In addition, the protein expression of anti-apoptotic genes, such as XIAP, survivin, Bcl-xL and Bcl-2, was inhibited by thymoquinone in breast cancer cells and breast tumor xenograft [103].

### 3.4. Pancreatic Cancer

Pancreatic cancer cells apoptosis was increased and tumor growth was synergistically inhibited by thymoquinone combined with gemcitabine both in vitro and in vivo via modulating multiple molecular signaling targets, such as suppressing Notch1 and Notch intracellular domain (NICD), up-regulating PTEN (phosphatase and tensin homolog deleted on chromosome ten), and inactivating Akt/themammaliantargetofrapamycin (mTOR)/S6 signaling pathways. The combination treatment down-regulated anti-apoptotic factors including Bcl-2, Bcl-xL and XIAP, up-regulated activation of pro-apoptotic molecules including caspase-3, caspase-9 and Bax, and increased release of cytochrome c. Thymoquinone pretreatment following gemcitabine treatment synergistically caused an increase in pancreatic cancer cells apoptosis and tumor growth inhibition both in pancreatic cancer cells in vitro and in PANC-1 cells orthotopic xenograft in vivo [107].

### 3.5. Hematopoietic Tumor

Apoptosis was induced by thymoquinone resulting from mitochondrial dysfunction in an acute lymphocyte leukemic cell line (lymphatic). Bcl-2 was down-regulated and Bax was up-regulated accompanied with thymoquinone-induced apoptosis with cell death-transducing signals [108]. Besides, thymoquinone increased early apoptosis, down-regulated the anti-apoptotic protein Bcl-2, and up-regulated the apoptotic protein Bax, showing high toxicity against murine leukemia cells (lymphatic) [109].

### 3.6. Colorectal Cancer

Both pre-treatment and post-treatment of thymoquinone could reverse 1,2-dimethyl-hydrazine (DMH)-induced oxidative stress at initiation and established histological changes and tumor development [110]. In another study, tumor growth in ApcMin (Min, multiple intestinal neoplasia) mice was interfered by thymoquinone through inducing tumor-cell specific apoptosis and modulating Wnt signaling via activation of glycogen synthase kinase (GSK)-3β, indicating *Nigella sativa* oil (or thymoquinone) might be useful as nutritional supplement in familial adenomatous polyposis [111]. Moreover, thymoquinone blocked STAT3 signaling via inhibition of Janus kinase (JAK) 2- and Src-mediated phosphorylation of EGFR tyrosine kinase, thus inducing apoptosis in human colon cancer cells [112]. Besides, thymoquinone led to caspase-independent, autophagic cell death via mitochondrial outer membrane permeability and activation of c-Jun *N*-terminal kinase (JNK) and p38 in irinotecan-resistant LoVo colon cancer cells [113]. In addition, in a xenograft model of HCT116 colon cancer cells, thymoquinone significantly inhibited the growth of the tumor cells [114].

### 3.7. Other Cancers

The anti-tumor activity of thymoquinone in oral cancer might be attributed to the down-regulation of p38β MAPK [115]. Besides, in head and neck squamous cell carcinoma, thymoquinone induced apoptosis involving an increase in Bax expression and caspase-9 activation, and induced autophagy which depended on increasing levels of autophagic vacuoles and LC3-II proteins, the specific autophagy markers [116]. Moreover, apoptosis was induced by thymoquinone markedly in two human cervical cell lines, such as Siha and C33A. Thymoquinone-induced apoptosis in Siha cells was through p53-dependent pathway, whereas apoptosis in C33A cells was associated with the activation of caspase-3 [117,118]. Similarly, thymoquinone also played a role in treatment of glioblastoma [119], melanoma [120], human T-cell leukemia virus-I-negative T-cell lymphomas [121] and osteosarcoma [122].

## 4. Ginger

**Figure 3:**
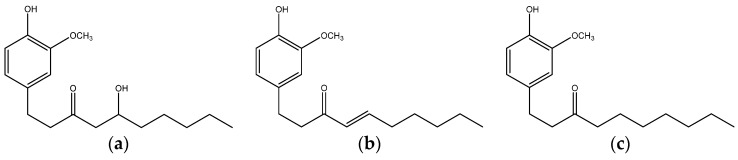
Structures of 6-gingerol (**a**); 6-shogaol (**b**) and 6-paradol (**c**).

Ginger (*Zingiber officinale*), a common spice in foods and beverages worldwide, is rich in several bioactive phenolics, including non-volatile pungent compounds such as gingerols, paradols and shogaols [123] (Figure 3), which possess antioxidant, anti-inflammatory, antifungal, anti-mycobacterial, and anticarcinogenic proprieties [124,125,126]. Also, ginger leaf has long been used as a vegetable, tea and herbal medicine [127].

### 4.1. Breast Cancer

Cancer development and progression could be decreased by 6-shogaol via inhibiting the production of inflammatory mediator chemokine (C-C motif) ligand 2 (CCL2), derived from breast tumor-associated dendritic cells (TADCs) [128]. Moreover, ginger supplementation was found to increase adiponectin, NO and GPx, and reduce MDA in obese women diagnosed with breast cancer [129]. Besides, another clinical trial showed inhaled ginger aromatherapy might be a complementary therapy for chemotherapy-induced nausea and vomiting in women with breast cancer [130].

### 4.2. Colorectal Cancer

6-Gingerol inhibited cell proliferation and induced apoptosis in colon cancer cells, but not in normal colon cells, which was associated with inhibition of ERK1/2/JNK/AP-1 pathway [131]. Besides, cysteine-conjugated shogaols, the major metabolites of shogaols in human body, exhibited similar toxicity towards human colon cancer cells [132]. In addition, the cell viability was reduced and apoptosis was induced in human colorectal cancer cells by the extracts of ginger leaf dose-dependently, and the effects resulted from activation of ATF3 promoter and following increase of ATF3 expression through ERK1/2 activation [127].

Several clinical studies researched the effects of ginger on colorectal cancer. In a pilot study, in people at increased risk of colorectal cancer, proliferation in the normal-appearing colorectal epithelium was reduced, and apoptosis and differentiation were increased by ginger [133]. Elevated tissue levels of PGE-2, whose production is regulated by COX-1 and NAD^+^-dependent 15-hydroxyprostaglandin dehydrogenase (15-PGDH), are an early event in colorectal cancer. After ginger consumption, colonic COX-1 protein expression in participants at increased risk for colorectal cancer was significantly reduced, but not in participants at normal risk of colorectal cancer. The 15-PGDH protein expression in either increased or normal-risk participants was not changed [134]. In another study focusing on PGE-2, there was a significant decrease in arachidonic acid after ginger treatment in the subjects at normal risk for colorectal cancer. In this way, ginger inhibited COX, and decreased the incidence and multiplicity of adenomas as well as PGE-2 concentrations [23].

### 4.3. Prostate Cancer

Whole ginger extract (GE) modulated cell-cycle and apoptosis regulatory molecules, impaired reproductive capacity, perturbed cell-cycle progression, and induced apoptosis in human prostate cancer cells. Tumor tissue from GE-treated mice was suppressed, and GE did not exhibit any detectable toxicity in normal tissues [123]. Besides, combination of GE and its constituents (in particular, 6-gingerol) led to remarked enhancement of GE’s antiproliferative activity [135]. In addition, constitutive and interleukin (IL)-6-induced STAT3 activation were reduced, and constitutive and TNF-α-induced NF-κB activity were inhibited by 6-shogaol in human (LNCaP, DU145, and PC3) and mouse (HMVP2) prostate cancer cells. The expression of several STAT3 and NF-κB-regulated target genes and apoptosis regulatory genes was decreased by 6-shogaol. 6-Shogaol was more effective than two other compounds, 6-gingerol and 6-paradol, in ginger at reducing survival of prostate cancer cells [136].

### 4.4. Other Cancers

6-Paradol is a pungent phenolic bioactive component of ginger. DMBA-induced neoplastic changes in male golden Syrian hamsters were reversed by oral administration of 6-paradol, and expression of apoptosis associated genes (p53, Bcl-2, caspase-3 and TNF-α) was also improved [137]. In addition, 6-shogaol possessed cytotoxicity on human lung cancer A549, by inhibiting the production of TADCs (tumor-associated dendritic cells)–derived CCL2 [128]. Moreover, ginger might be used for the treatment of melanoma [138] and glioblastoma [139].

## 5. Garlic

**Figure 4:**
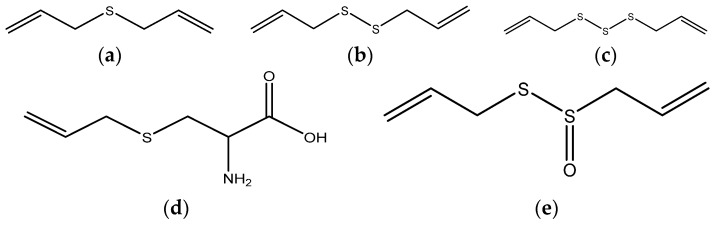
Structures of diallyl sulfide (**a**); diallyl disulfide (**b**); diallyl trisulfide (**c**); *S*-allyl mercaptocysteine (**d**) and allicin (**e**).

Garlic (*Allium sativum*) is a widely used spice, and also a traditional remedy for a variety of ailments. Garlic possesses cancer-preventive potential and significant enhancing effects on the immune system. The potential anticancer effects of garlic are attributed to its metabolic byproducts, organosulfur components [140,141]. Natural organosulfur compounds exhibit antioxidant and chemo-sensitization properties, and they draw wide attention, such as diallyl sulfide, diallyl disulfide, diallyl trisulfide, diallyl tetrasulfide, *S*-allyl mercaptocysteine, and allicin [142] (Figure 4).

### 5.1. Breast Cancer

Breast cancer growth was inhibited by miR-34a, and the anti-tumor effect of diallyl disulfide was augmented by miR-34a. SRC expression was inhibited by miR-34a, which resulted in the suppression of the SRC/Ras/ERK pathway. Diallyl disulfide up-regulated expression of miR-34a in MDA-MB-231 cells, so it possessed cytotoxicity effects [143]. Moreover, diallyl sulfide inhibited diethylstilbestrol-induced DNA damage in human breast epithelial cells (MCF-10A), and reduced lipid peroxidation [144]. Besides, after treatment with *S*-allyl mercaptocysteine, cell growth was inhibited in human breast cancer cell lines MCF-7 (ER^+^) and MDA-MB-231 (ER^−^) via inducing cell cycle arrested in G_0_/G_1_ phase. Accompanied with the cell cycle arrest, apoptosis was promoted. The mitochondrial apoptotic pathway was triggered by activating Bax, decreasing expression of Bcl-2 and Bcl-XL, and subsequent activating caspase-9 and caspase-3 [145]. In addition, attenuating or blocking the release of CCL2 resulted in preventing cancer-associated inflammation. Diallyl disulfide reversed TNF-α-induced CCL2 release in human breast tumor (MDA-MB-231) cells [146]. Similarly, estrogen receptor-α (ER-α) activity was inhibited by diallyl trisulfide in human breast cancer cells. ER-α protein was down-regulated after exposure to diallyl trisulfide in MCF-7 and T47D cells, accompanied with a decrease in nuclear levels of ER-α protein, but were not affected in the presence of 17β-estradiol [147].

In experimental animals, allicin enhanced chemotherapeutic response, and ameliorated hepatic injury induced by tamoxifen, which was widely used for treatment of hormone-dependent breast cancer [148]. In another animal study, treatment with diallyl disulfide significantly reduced tumor volume and weight, and increased apoptosis in MDA-MB-231 xenograft mice by decreasing the expression of active β-catenin [149].

### 5.2. Upper Digestive Tract Cancer

Cell cycle redistribution plays an important role in diallyl disulfide-modulated anticarcinogenic effects in human gastric cancer cells, so the checkpoint kinases (Chk1 and Chk2) were further studied. The results showed that diallyl disulfide-mediated G_2_/M arrest was regulated by Chk1 through ATR/Chk1/CDC25C/cyclin B1 [150]. Similarly, cell viability was markedly reduced by diallyl disulfide in esophageal squamous cell carcinoma cells dose- and time-dependently. G_2_/M phase arrest was induced by diallyl disulfide by decreasing cyclin B1, CDC2, p-CDC2 and cdc25c, and activating the p53/p21 pathway. Apoptosis was induced by diallyl disulfide through activation of caspases, alteration of Bax/Bcl-2 balance and suppression of the MEK-ERK pathway [151]. The results were also verified in human esophageal carcinoma ECA109 cells and in mice [152]. In addition, apoptosis of MGC803 human gastric carcinoma cells was markedly increased by allicin, accompanied with an enhancement of expression levels of cleaved caspase-3, and the protein expression levels of p38 were also increased [153]. Notably, epidemiological studies also showed that garlic intake resulted in reduced risk of gastric cancer [154,155].

### 5.3. Colorectal Cancer

Garlic contained natural organoselenium compounds such as selenomethionine and se-methyl-l-selenocysteine (MseC), which possessed lower toxicity and better anticancer activities than inorganic Se. The 80% apoptosis in colo 205 cells was caused by MseC, which was involved in caspase activation, the extrinsic apoptotic pathway, and the regulation of ER-stress-induced apoptosis [156]. Moreover, aged garlic extract (AGE) is produced from fresh garlic for more than 10 months. The number of ACF was decreased by AGE. The proliferative activities in adenoma and adenocarcinoma lesions were suppressed, without effects on normal colon mucosa. Cell cycle progression, cyclin B1 and cdk1 expression were down-regulated via inactivation of NF-κB in the human colorectal cancer cells [157].

### 5.4. Hematopoietic Tumor

According to Suda et al., heat shock protein 27 (HSP27) was one of the molecular targets of diallyl trisulfide in human leukemic cell line U937 (myeloid) [158]. In another study, *N*-benzyl-*N*-methyldecan-1-amine (NBNMA) was isolated from garlic cloves. Cell cycle arrest at the G_2_/M phase and apoptotic cells were induced by NBNMA in U937 cells. The expression of regulator genes of G_2_/M phase progression, cyclin dependent kinase (Cdk) 2 and CDC2 was suppressed, and the expression of the Cdk inhibitor p21WAF1/CIP1 was enhanced. Caspase-8 and caspase-9 were also activated [159].

### 5.5. Other Cancers

The compounds extracted from garlic play an important role in the treatment of many other cancers. For example, z-ajoene, a garlic-derived compound, was a potential candidate for the treatment of glioblastoma multiforme by specifically targeting glioblastoma multiforme cancer stem cells [160]. In addition, thiacremonone is a novel sulfur compound generated from high-temperature-high-pressure-treated garlic. The growth of lung tumor cells was inhibited by thiacremonone via inhibiting Gpx activity of peroxiredoxin 6 through interaction [161]. Moreover, diallyl trisulfide possessed cancer-preventive effects in osteosarcoma, pancreatic cancer, and bladder cancer [162,163,164]. Besides, *S*-allylcysteine might be useful in treatment of ovarian cancer [165], and (*S*)-*N*-*trans*-feruloyloctopamine could be used for treatment of melanoma [166].

### 5.6. Other Allium Genus Spices

Onion (*Allium cepa*) and scallion (*Allium fistulosum*) were also included in *Allium* genus, which showed cancer-preventive effects, attributed to sulfur-containing compounds [141]. Moreover, there are also other constituents in onion, such as quercetin and fisetin, possessing anticancer effects. For instance, quercetin-induced inhibition of migration and invasion of human oral cancer cells was attributed to down-regulating PKC and RhoA by blocking MAPK and PI3K/AKT signaling pathways and NF-κB and uPA, thus suppressing MMP-2 and MMP-9 signaling [167]. Besides, fisetin showed cancer-preventive effects via modulating the PI3K/Akt/mTOR pathway in cancer cell models [168], and in animal models [169]. Similarly, selenomethionine and Se-methyl-l-selenocysteine were also found in onion, and the effect was like in garlic [156]. In addition, red onion might decrease the risk of ovarian cancer [170,171].

In another study, scallion extracts showed significant suppression of colon tumor growth in mice, through inhibiting the key inflammatory markers COX-2 and iNOS, and suppressing the expression of various cellular markers involved in tumor apoptosis, proliferation, angiogenesis and invasion [172].

## 6. Saffron

**Figure 5:**
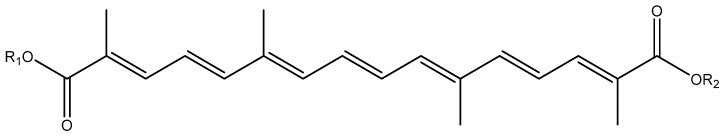
Structures of crocin (R_1_ = R_2_ = gentiobiosyl) and crocetin (R_1_ = R_2_ = H).

Saffron (*Crocus sativus*), the dried, dark red flower, is harvested from stigmas of the plant. It was one of the most expensive spices in the world, and was used as a spice for flavoring and coloring food, and as an herbal plant in folk medicine. A number of studies showed that saffron possessed anticancer effects which was attributed to the bioactive compounds it contained, such as crocin and crocetin (Figure 5). The compounds were abundant in saffron, and they induced apoptosis and inhibited cell proliferation [173,174].

### 6.1. Lung Cancer

According to Samarghandian et al., cell viability in cultured human alveolar basal epithelial carcinoma cells was inhibited by the ethanolic extract of saffron, which might be a potential chemotherapeutic agent in lung cancer [175]. In their further study, the cytotoxicity activity of the aqueous extract of saffron was through inducing apoptosis and inhibiting cell proliferation via activating caspase-dependent pathways in the A549 cells [176].

### 6.2. Digestive System Cancer

In both human adenocarcinoma gastric cancer cells and rat model of gastric cancer, crocetin induced apoptosis, suppressed Bcl-2 and up-regulated Bax expression in gastric adenocarcinoma cells. Crocetin also reversed 1-methyl-3-nitro-1-nitrosoguanidine-induced changes in serum antioxidant activity and lactate dehydrogenase [177]. Moreover, crocin induced apoptosis in the gastric adenocarcinoma cells. The Bax/Bcl-2 ratio was increased, which indicated that apoptosis was stimulated by crocin, and crocin possessed anticancer effect [178]. Additionally, crocin induced an autophagy-independent classical programmed cell death in colon cancer cells [179].

### 6.3. Reproductive System Cancer

Saffron extract and its major constituent crocin reduced cell proliferation in malignant prostate cancer cell lines. Saffron extract and crocin could down-regulate the expression of Bcl-2, and up-regulate the expression of Bax [180]. Besides, compared to crocin and saffron, crocetin possessed stronger antitumor effects, which was confirmed by reducing *N*-cadherin and β-catenin expression and increasing expression of E-cadherin. Prostate cancer cell invasion and migration were inhibited by saffron, crocetin and crocin via down-modulating metalloproteinase and urokinase expression/activity suggesting that these agents might affect metastatic processes [181]. Moreover, crocin could remarkably suppress the growth of ovarian cancer HO-8910 cells, arrest the cells in the G_0_/G_1_ phase, and promote cell apoptosis via increasing p53 and Fas/APO-1 expression and activating caspase-3-regulated apoptotic pathway [182].

### 6.4. Other Cancers

Crocetin is a carotenoid dicarboxylic acid which is abundant in saffron. The proliferation and invasion in the highly invasive MDA-MB-231 cells were markedly inhibited by crocetin. MMPs were related to cancer invasiveness and metastasis. Crocetin significantly suppressed the gene and protein expression of pro-MT1-MMP and pro-MT2-MMP, and decreased pro-MMP-9 activity and pro-MMP-2/MMP-2 protein levels [183]. Besides, crocin displayed mild cytotoxic effects on leukemic cells, which was modulated by increased DNA fragmentation [184]. Moreover, crocin and safranal modulated cytotoxic response to K-562 human chronic myelogenous leukemia cells (myeloid) [185]. In addition, crocin was a potential anticancer agent of osteosarcoma [186].

## 7. Black Pepper and Piperine

**Figure 6:**
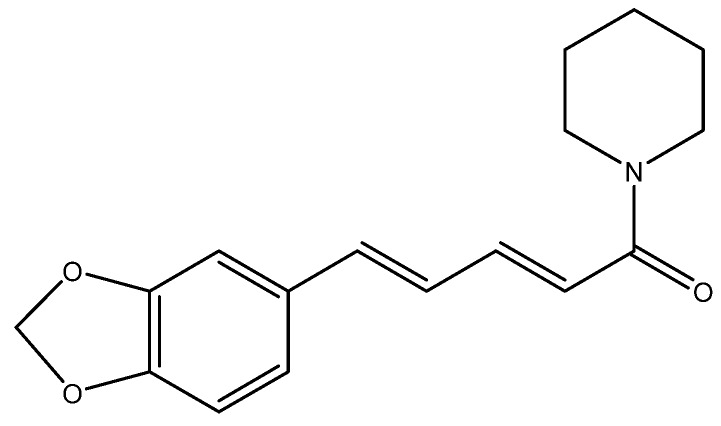
Structure of piperine.

Black pepper (*Piper nigrum*) is a widely consumed spice, which is also an herb commonly used in folk medicine. Piperine (Figure 6), a major alkaloid constituent of black pepper, exerts antitumor activities in a variety of cancers.

### 7.1. Breast Cancer

Piperine inhibited proliferation and induced apoptosis via activating caspase-3 and PARP cleavage. EGF-induced MMP-9 expression was suppressed by piperine via interfering with ERK1/2, p38 MAPK, leading to a reduction in migration. The sensitization of human epidermal growth factor receptor (HER) 2-overexpressing breast cancer cells to paclitaxel was augmented by piperine [187]. In addition, among the 55 compounds deprived from natural plants which were screened in the study, piperine was the most potent adjuvant at enhancing the efficacy of TNF-related apoptosis-inducing ligand-based therapies in triple negative breast cancer cells, probably modulated via inhibiting survivin and p65 phosphorylation [188]. Besides, the growth of triple negative breast cancer cells and hormone-dependent breast cancer cells was both inhibited. Piperine also induced apoptosis in triple negative breast cancer cells via the mitochondrial pathway. A combination of piperine and γ radiation exerted more cytotoxicity for triple negative breast cancer cells than γ radiation alone. Piperine inhibited the growth of triple negative breast cancer xenografts in immune-deficient mice [189].

### 7.2. Prostate Cancer

The proliferation of LNCaP, PC-3, 22RV1 and DU-145 prostate cancer cells was inhibited by piperine. Piperine treatment remarkably suppressed both the androgen dependent and androgen independent tumor growth in nude mice model xenotransplanted with prostate cancer cells [190]. Moreover, piperine treatment dose-dependently inhibited the proliferation, induced cell cycle arrest at G_0_/G_1_, down-regulated cyclin D1 and cyclin A, and promoted autophagy in LNCaP and PC-3 cells [191]. Besides, a combination of piperine and docetaxel remarkably improved the anti-tumor efficacy of docetaxel in a xenograft model of human castration-resistant prostate cancer [192].

### 7.3. Colorectal Cancer

Piperine inhibited the metabolic activity of HRT-18 human rectal adenocarcinoma cells indicating a cytostatic effect. Piperine inhibited cell cycle progression and induced apoptosis [193]. Furthermore, piperine inhibited HT-29 colon carcinoma cell proliferation by causing G_1_ phase cell cycle arrest. Piperine induced loss of mitochondrial membrane integrity and cleavage of poly (ADP-ribose) polymerase-1. The colony formation and the growth of HT-29 spheroids were inhibited [194].

### 7.4. Other Cancers

Piperine-modulated ROS induced DNA damage and activation of Chk1, thus leading to G_1_ cell cycle arrest and apoptosis in melanoma cells [195]. Similarly, piperine was also a promising therapeutic agent in the treatment of osteosarcoma [196].

## 8. Red Chili Pepper and Capsaicin 

**Figure 7:**
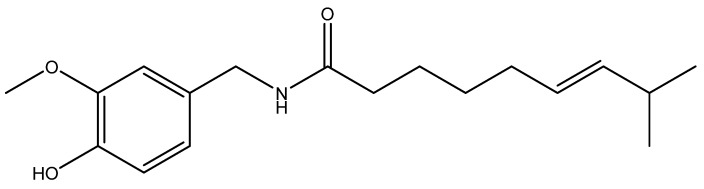
Structure of capsaicin.

Red chili pepper (*Capsicum annum*) is a widely consumed spice throughout the world. Capsaicin (Figure 7), the most abundant pungent ingredient of red chili peppers, exerts potent anticancer effect in various human malignancies [197].

### 8.1. Lung Cancer

Capsaicin exclusively targeted angiogenesis via down-regulating VEGF in non-small cell lung carcinoma cells [198]. Besides, capsaicin exhibited pro-apoptotic activity via down-regulating transient receptor potential vanilloid (TRPV) receptor in human small cell lung cancer cells [199]. In addition, capsaicin induced oxidative DNA damage in non-small cell lung carcinoma cells, which was related to the p53/miR-34a regulatory axis [200]. Besides, capsaicin exerted anti-proliferative activity against human small cell lung cancer in cell culture and nude mice models via the E2F pathway [201].

### 8.2. Breast Cancer

As previously mentioned, TRPV superfamily was modulated by capsaicin. However, the over-expression or activation of TRPV1 was not associated with capsaicin-induced proliferation in MCF-7 breast cancer cells. Up-regulation of c-Fos and RIP3 was responsible for capsaicin-induced cell death [202]. Moreover, the cytotoxic effects of water extract of chili pepper seeds were investigated. The extract contained phenols but no capsaicinoids. The proliferation of breast cancer cells was suppressed, the expression levels of E-cadherin were increased, and the secretion of MMP-2 and MMP-9 was decreased by the extract [203]. Besides, activation of ERK and expression of HER-2 and cyclin D1 were decreased, while caspase activity and PARP cleavage products were increased in tumors of capsaicin-treated mice [204].

### 8.3. Gastric Cancer

According to Meral et al., capsaicin possessed significant cytotoxic effects, and decreased inhibitory concentration IC_50_ value of 5-flourouracil [205]. In addition, capsaicin inhibited the proliferation of human gastric cancer cells (AGS cells) and induced apoptosis, via increase of cleaved caspase-3, reduction of Bcl-2, and decrease in the expression of phosphorylated ERK 1/2, p38 MAPK or JNK [206].

### 8.4. Cholangiocarcinoma

Capsaicin induced anti-migration and anti-invasion effects in cholangiocarcinoma cells through inhibition of NF-κB p65, resulting in subsequent suppression of MMP-9 expression [207]. In another study, proliferation, migration and invasion of human cholangiocarcinoma cells were all effectively inhibited by capsaicin, which was associated with regulating the Hedgehog signaling pathway [208].

### 8.5. Prostate Cancer

The chemopreventive potential of capsaicin on prostate cancer was investigated in the transgenic adenocarcinoma of the mouse prostate model. The capsaicin-treated mice showed a trend of lower-grade disease with better differentiated adenocarcinoma, compared to the control group. The metastatic burden in prostate tumors was reduced, and the invasion and migration capacity of PC3 cells were suppressed in vitro [209]. Moreover, capsaicin sensitized human prostate cancer cells to radiotherapy via inhibiting NF-κB signaling. A combination of oral administration of capsaicin and radiotherapy led to a more marked growth delay and reduction than capsaicin or radiotherapy alone in vivo [210]. In addition, when given orally, capsaicin significantly slowed the growth of PC-3 prostate cancer xenografts in mice as measured by size and weight [211].

### 8.6. Other Cancers

Capsaicin induced apoptosis in acute lymphoblastic leukemia cells (lymphatic), which was associated with down-regulation of cell signaling pathways [212]. Besides, the anticancer effect of capsaicin on pancreatic cancer [213,214], colorectal cancer [215], and bladder cancer [216].

## 9. Rosemary

**Figure 8:**
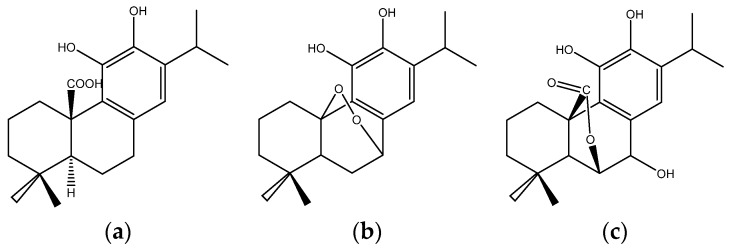
Structures of carnosic acid (**a**); carnosol (**b**) and rosmanol (**c**).

Rosemary (*Rosmarinus officinalis*) is a popular spice widely used in Western diets, especially in the “Mediterranean Diet”, which showed preventive effects of cardiovascular disease, diabetes and various solid cancers. Carnosic acid, carnosol and rosmanol (Figure 8) may be the active components contained in rosemary responsible for its anti-cancer activity [217].

### 9.1. Colorectal Cancer

The supercritical fluid rosemary extract (SFRE, the extract obtained from rosemary leaves by supercritical fluid extraction) alone or combined with 5-FU, exerted a cytotoxic effect on colon cancer cells. 5-FU-resistant cells were sensitized by the extract via down-regulating TYMS, TK1, and enzymes related to 5-FU resistance [218]. In addition, carnosic acid-rich rosemary extract showed anticancer properties in colon and pancreatic cancer, and GCNT3 expression was involved in its antitumor mechanism [219]. Moreover, carnosol significantly reduced cell viability and induced apoptosis in human colon cancer via generating ROS, inducing p53, activating caspases and inhibiting the STAT3 signaling pathway [220].

### 9.2. Other Cancers

SFRE exhibited antitumor activity against breast cancer cells, and down-regulated estrogen-dependent-α and HER2 receptors. The effect of breast cancer chemotherapy was also significantly enhanced by SFRE [221]. In addition, carnosic acid inhibited cell migration and suppressed the adhesion via inhibition of the epithelial-mesenchymal transition in B16F10 cell migration [222]. Besides, carnosic acid markedly induced TRAIL-mediated apoptosis in human renal carcinoma, human hepatocellular carcinoma, and human breast carcinoma cells, through down-regulating c-FLIP and Bcl-2 expression, and up-regulating ER stress-mediated DR5, Bim, and p53 up-regulated modulator of apoptosis (PUMA) expression at the transcriptional levels, without affecting normal cells [223]. Moreover, rosemary extract decreased androgen receptor expression and suppressed tumor growth in human prostate cancer cell lines, such as 22Rv1 and LNCaP [224]. Additionally, carnosol induced apoptosis by affecting the redox status and decreasing glutathione in the adult T-cell leukemia/lymphoma cells (lymphatic) [225]. In addition, carnosic acid significantly induced autophagic cell death in HepG2 cells, which was associated with inhibition of the Akt/mTOR pathway [226].

## 10. Other Spices

Clove (*Syzygium aromaticum*), the sun-dried unopened flower bud from the plant, has been used as a common spice and a traditional Chinese medicine. It exerts antiseptic, antibacterial, antifungal, and anticancer properties. Eugenol was a major component in clove and several other spices such as basil, cinnamon, and bay leaves. Oleanolic acid is also one of the ingredients of clove extract attributed to its antitumor activity. For instance, eugenol reduced ATP utilization and oxidative stress, and increased the polyamines and glycolytic metabolites in oral squamous cell carcinoma cells [227]. In addition, eugenol also exerted cancer-preventive properties in breast cancer both in vitro and in vivo through targeting the E2F1/survivin pathway [228], and eugenol exerted pro-apoptotic and anti-inflammatory properties in human cervical cancer cells [229]. Clove might be a potential therapeutic agent against digestive system cancers. In the study of Dwivedi et al., among water, ethanol and oil extracts of clove, the oil extract exerted maximal cytotoxic activity. Cell growth was inhibited by the oil extract of extract in TE-13 esophageal cancer cell lines [230]. Moreover, clove may represent a novel therapeutic herb for the treatment of colorectal cancer, and oleanolic acid was one of the components in ethyl acetate extract of cloves responsible for its antitumor activity [231]. Similarly, eugenol also contributed to treatment against liver cancer [232].

Galangal (*Alpinia officinarum*) is a traditional oriental spice, and used in folk medicine. Galangin, a flavonol derived from galangal exerted anticancer effects on several cancers, including melanoma, hepatoma, and colon cancer cells. For instance, apoptotic pathways in cancer cells might be activated by prolonged endoplasmic reticulum stress. Galangin induced endoplasmic reticulum stress in hepatocellular carcinoma cells (HepG2, Hep3B and PLC/PRF/5 cells) [233]. In addition, galangin also induced autophagy in hepatocellular carcinoma cells, via activating the TGF-β receptor/Smad pathway [234]. Moreover, MMP-9 degrades type IV collagen in the basement membrane and plays crucial roles in several pathological implications, including tumorigenesis and inflammation. In this study, phorbol-12-myristate-13-acetate-induced MMP-9 expression in human fibrosarcoma HT-1080 cells was suppressed by galangin, via blocking activation of NF-κB and AP-1 [235]. Besides, galangin also exerted anticancer effects in colon cancer cells [236] and in melanoma cells [237].

Coriander (*Coriandrum sativum*) is used as a common culinary spice and medicinal herb of the Apiaceae family. Ethyl acetate extract of coriander roots possessed antiproliferative activity on MCF-7 cells, inhibited DNA damage and prevented MCF-7 cell migration induced by H_2_O_2_ [238]. Besides, linalool, abundant in coriander, is one of the active components responsible for its anticancer effect. The anticancer effect of linalool was through inducing oxidative stress. Linalool exhibited pro-oxidant effect in tumor tissue and modulated the proliferation of spleen cells in tumor-bearing mice, without affecting the normal cells [239].

Wasabi (*Wasabia japonica*) is a typical Japanese spice, which belongs to the family *Brassicaceae* and contained various isothiocyanates (ITCs). 6-(Methylsulfinyl) hexyl isothiocyanate (6-MITC), an aromatic component isolated from wasabi, 6-MITCs induced apoptosis in breast cancer cells via inhibiting NF-κB and modulating the PI3K/AKT pathway [240].

Cinnamon (*Cinnamomum cassia*), a traditional oriental medicinal herb, is also widely used as a spice. Cinnamaldehyde, the bioactive component isolated from the stem bark of cinnamon, exerted anticancer activity against various cancers. For instance, the volume of tumors and the number of new vessels in melanoma cells were decreased after administration of the cinnamaldehyde in mice. Cinnamaldehyde suppressed the expression of HIF-α and VEGF in the melanoma [241]. Moreover, cinnamon polyphenols might exhibit neuroprotective effects in C6 glioma cells via regulating Bcl-2 and augmenting SIRT1 expression [242]. Besides, cinnamaldehyde combined with chemotherapeutic agents (5-FU, OXA) exerted a synergistic effect on cytotoxicity in colorectal carcinoma cells. Expression of BRCA1, TOPO1, ERCC1 and TS mRNA was suppressed by cinnamaldehyde [243]. In another study, 2′-hydroxycinnamicaldehyde, a compound found in cinnamon, was a potent anticancer agent as a result of direct targeting of the Pim-1 kinase [244]. In addition, cinnamon aqueous extract induced apoptosis in the human myelocytic leukemia cell line (myeloid) [245].

Oregano (*Origanum vulgare*) is a widely used spice in the “Mediterranean Diet” and contains carvacrol, thymol and many other anticancer components. For instance, β-caryophyllene oxide, a sesquiterpene isolated from the essential oils of medicinal plants including oregano, showed an anticancer effect through blocking the STAT3 activation pathway in cancer cells [246]. Moreover, carvacrol induced apoptosis via the mitochondrial apoptotic pathway and the MAPK and PI3K/Akt signaling pathways in colon cancer cells [247].

Cardamom (*Elettaria cardamom*), a dietary phytoproduct, is used as spice, and exerts cancer chemopreventive potential. Cardamom ingestion blocked NF-κB activation and down-regulated cyclo-oxygenase-2 expression in skin papillomas in DMBA-treated mice [248]. Besides, cardamom reduced B(α)P-induced forestomach tumor incidence and significantly enhanced the hepatic activities in mice [249].

Besides, the anticancer potential of some spices less widely used, such as Fenugreek (*Trigonella foenum graecum*) [250], red yeast rice [251], *Piper sarmentosum* [252] and *Murraya koenigii* [253], were also mentioned in the literature.

The most common sites of cancer diagnosed in 2012 were lung, liver, breast, stomach, colorectum, cervix, and prostate [24], and these cancers draw wide attention in the research of spices (Table 1).

## 11. Bioavailability of Active Compounds from Spices

The compounds derived from spices are usually with relative low bioavailability, such as curcumin, thymoquinone, piperine and capsaicin [254,255,256,257]. Many methods have been proposed to enhance the bioavailability of these compounds in vivo. For instance, the bioavailability of curcumin could be enhanced by molecular complexation of curcumin with pH sensitive cationic copolymer [258]. Moreover, nanocarrier loading and microparticles containing curcumin could improve bioavailability of curcumin [259,260]. Additionally, a novel curcumin analog showed anti-tumor activity and improved bioavailability [261]. As to thymoquinone, its absorption after administration was relatively slow [256], and novel analogs of thymoquinone might possess superior bioavailability and anti-tumor activity [262]. In a study about ginger, a suitably designed multiparticulate system containing ginger extract improved the therapeutic efficiency of colon cancer [263]. In addition, capsaicin-loaded microemulsion and liposomal nanoformulation both enhanced oral bioavailability [264,265,266,267]. As for piperine, a self-emulsifying drug delivery system could enhance oral bioavailability of piperine [268].

## 12. Side Effects of Active Compounds from Spices

Spices are commonly consumed in human diets, and most varieties at appropriate doses are safe to humans. Purified compounds are separated from spices for treatment of diseases. A few studies reported the side effects of these bioactive compounds when they were used for treatment of cancers. Some compounds might produce toxic and carcinogenic effects under specific conditions. For example, carcinogenic and toxic effects of curcumin were found in a long term study (2 years) in rats and mice, while no carcinogenic effect was observed in short term studies (3 months) [269]. Curcumin might induce DNA damage in normal cells in the presence of Cu^2+^ in vitro and in vivo [270,271]. In addition, safrole, a member of benzodioxoles, shows carcinogenic activities and is present naturally in essential oils of spices including black pepper, cumin, ginger, etc. Thermal treatments such as drying (70 °C, 30 min) or boiling (5 min) during cooking could decrease dose of safrole to a safer level [272]. Besides, several compounds with anti-platelet effects from garlic, black cumin, ginger, fenugreek and turmeric might lead to excess bleeding in patients with bone marrow suppression [273,274,275].

## 13. Conclusions

Several spices have exerted anticancer effects including lung, liver, breast, stomach, colorectum, cervix, and prostate cancers. Direct extract, essential oil, and compounds isolated from spices are commonly studied. Among the mentioned compounds, curcumin is most widely researched in the papers, which might be useful in prevention and treatment of a broad spectrum of cancers, and thymoquinone has attracted wide attention as well. Some spices’ compounds exert anticancer properties in both cells and animal models, suggesting they might be effective in human cancer. Several components of spices (especially ginger and garlic) show their anticancer effects in the digestive system, indicating these spices might be a healthy dietary means to prevent cancer directly. Some spices exerted their anticancer properties by inducing apoptosis, cell death and DNA damage, causing G_2_/M arrest, inhibiting tumorigenesis, proliferation, invasion, metastasis and migration. In addition, the anticancer properties of spices against breast and prostate cancer are related to regulating hormones or hormone receptors, including estrogen receptor and androgen receptor. Some spices could also sensitize cancer cells to radiotherapy and chemotherapeutic drugs, such as 5-FU and gemcitabine. The doses to achieve equivalent cancer control of radiation or the chemotherapy drugs were lowered by combined treatment of spices, thus minimizing the adverse effects to normal tissues. The efficacy of existing chemotherapeutic agents and radiotherapy were enhanced, indicating that combined treatment is a potential therapeutic strategy for cancers. In a word, spices are promising sources of adjuvant therapy of cancer. In the future, more anticancer bioactive components in spices should be separated and identified, and the mechanisms of action should be further explored.

## Figures and Tables

**Table 1:** The anticancer activities of spices.

Sites	Spices	Constituents	Anticancer Effects	References
Lung	Turmeric	Curcumin	Inducing apoptosis and DNA damage; inhibiting proliferation, migration, and the growth of cancer; decreasing cell growth and viability; inhibiting expression of DNA-repair-associated proteins	[34,35,36,37,38,39,40,41,42]
	Black cumin	Seed extract and seed oil; Thymoquinone	Reducing viability of human lung cancer; inhibiting proliferation, migration, and invasion of lung cancer cells	[98,99,100]
	Ginger	6-Shogaol	Decreasing tumorigenesis and the metastasis	[128]
	Garlic	Thiacremonone	Inhibiting tumor growth	[161]
	Saffron	Ethanolic extract, aqueous extract	Inducing cell death and apoptosis, inhibiting the cell proliferation	[175,176]
	Red chili pepper	Capsaicin	Restraining angiogenesis, inducing apoptosis and oxidative DNA damage	[198,199,200,201]
Liver	Turmeric	Curcumin	Inhibiting the growth of hepatoma cells, inhibiting and reversing diethylnitrosamine-induced hepatocarcinogenesis	[44,45,46]
	Black cumin	Thymoquinone	Inhibiting cell proliferation	[101,102]
	Rosemary	Carnosic acid	Sensitizing TRAIL-mediated apoptosis, inducing autophagic cell death	[223,226]
	Clove	Eugenol	Improving the xenobiotic-metabolizing systems	[232]
	Galangal	Galangin	Inhibiting proliferation of cancer cells,	[233,234]
Breast	Turmeric	Curcumin	Inhibiting MCF-7 breast carcinoma cells, cell invasion, and sensitizing cancer cells to retinoic acid	[43,49,50,51,52,53,54,55,56,57,58,59,60]
	Black cumin	Thymoquinone	Anti-proliferative and pro-apoptotic effects	[103,104,105,106]
	Ginger	6-Shogaol	Decreasing tumorigenesis and the metastasis	[128,130]
	Garlic	Diallyl disulfide, Diallyl sulfide, Diallyl trisulfide, *S*-allyl mercaptocysteine	Inhibiting proliferation, cell growth, and metastasis; inhibiting diethylstilbestrol induced DNA damage; inducing apoptosis; immunomodulation; inhibiting estrogen receptor-α activity	[143,144,145,146,147,148,149]
	Saffron	Crocetin	Inhibiting invasiveness	[183]
	Black pepper	Piperine	Inhibiting proliferation, the growth and motility of cells, inducing apoptosis, enhancing the efficacy of TRAIL-based therapy	[187,188,189]
	Red chili pepper	Capsaicin	Inducing cell death, inhibiting invasion and migration	[202,204]
	Rosemary	Supercritical fluid rosemary extract	Downregulating estrogen receptor-α and HER2 receptors, sensitizing TRAIL-mediated apoptosis	[221,223]
	Clove	Eugenol	Inducing apoptosis	[228]
	Coriander	Ethyl acetate extract	Inhibiting DNA damage and migration	[238]
	Wasabi	6-MITC	Inducing apoptosis	[240]
Stomach	Turmeric	Curcumin	Inhibiting proliferation and invasion, promoting apoptosis, suppressing lymphatic vessel density, inhibiting cell growth	[61,62,63,64,65]
	Garlic	Diallyl disulfide	Causing G_2_/M arrest, promoting apoptosis, suppressing xenograft tumors	[150,151,152,153,154,155]
	Saffron	Crocetin, crocin	antioxidant, anti-proliferative, and apoptotic activities	[177,178]
	Red chili pepper	Capsaicin	Inhibiting cell proliferation, inducing apoptosis	[205,206]
	Cardamom	Not mentioned	Inhibiting Benzo(α)Pyrene-induced forestomach papillomagenesis	[249]
Colorectum	Turmeric	Curcumin	Preventing aberrant crypt foci, inducing apoptosis, inhibiting cell growth	[66,67,68,69,70,71,72]
	Black cumin	Thymoquinone	Attenuating tumor development and growth, inducing apoptosis, inducing autophagic cell death	[110,111,112,113,114]
	Ginger	Ginger root/leaf extract, 6-gingerol, shogaols	Reducing cell viability and proliferation, inducing apoptosis	[23,127,131,132,133,134]
	Garlic	Se-Methyl-l-selenocysteine garlic extract	Inducing apoptosis, suppressing cell proliferation	[156,157]
	Onion	Se-Methyl-l-selenocysteine	Inducing apoptosis	[156]
	Scallion	Scallion extract	Inhibiting tumor growth	[172]
	Saffron	Crocin	Inducing apoptosis	[179]
	Black pepper	Piperine	Impairing cell cycle progression and inducing apoptosis	[193,194]
	Red chili pepper	Capsaicin	Inhibiting cell proliferation and inducing apoptosis	[215]
	Rosemary	Rosemary extract, carnosic acid, diterpenes	Sensitizing cancer cells to 5-FU, inhibiting cell migration, inducing apoptosis	[218,219,220]
	Clove	Clove extract	Inhibiting tumor growth and promoting cell cycle arrest and apoptosis	[231]
	Galangal	Galangin	Inducing cell death	[236]
	Cinnamon	Cinnamaldehyde	Regulating drug-metabolizing genes	[243]
	Oregano	Carvacrol	Inhibiting proliferation and induces apoptosis	[247]
Cervix	Turmeric	Curcumin	Eradicating HPV^+^ cancer cells without affecting non-cancerous tissue, inhibiting the proliferation and inducing apoptosis, inhibiting tumor growth and angiogenesis	[76,78,79]
	Black cumin	Thymoquinone, methanolic extract	Inducing apoptosis and inhibiting proliferation	[117,118]
	Clove	Eugenol	Enhancing the effect of gemcitabine, anticarcinogenic and anti-inflammatory activity	[229]
Prostate	Turmeric	Curcumin	Targeting AR and histone modification, inhibiting the proliferation and growth	[73,74,75]
	Ginger	Ginger extract, 6-shogaol, 6-gingerol and 6-paradol	Inducing apoptosis, inhibiting prostate cancer cell proliferation and growth	[123,135,136]
	Saffron	Saffron extract	Antiproliferative properties, inhibiting cell invasion and migration	[180,181]
	Black pepper	Piperine	Reducing the androgen dependent and androgen independent tumor growth, inhibiting proliferation	[190,191]
	Red chili pepper	Capsaicin	Reducing the metastatic burden, radio-sensitizing agent	[209,210]
	Rosemary	Rosemary extract	Promoting androgen receptor degradation and decreasing xenograft tumor growth	[224]

## Abbreviations

The following abbreviations are used in this manuscript:

ACF, aberrant crypt foci; AP-1, active protein-1; AFP, α feto-protein; AgNOR, silver-staining nucleolar organizer region; ALT, alanine amino transferase; AR, androgen receptor; AST, aspartate aminotransferase; Bax, Bcl-2 associated X protein; Bcl-2, B-cell lymphoma-2; Bcl-xL, B-cell lymphoma-xL; BRCA1, breast cancer susceptibility gene 1; CAT, catalase; CBP, cAMP response element-binding protein; COX, cyclooxygenase; DLEC1, deleted in lung and esophageal cancer 1; EMT, epithelial-mesenchymal transition; EGR, early growth response; DENA, diethylnitrosamine; EGFR, epidermal growth factor receptor; ERK, extracellular regulated protein kinase; 5-FU, 5-fluorouracil; GR, glutathione reductase; Gpx, glutathione peroxidase; 5-HETE, 5-hydroxyeicosatetraenoic acid; HIF, hypoxia-inducible factor; IL-2, interleukin-2; IL-6, interleukine-6; KLF4, Kruppel-like factor 4; LYVE, lymphatic vessel endothelial receptor; lncRNAs, long non-coding RNAs; MAPK, mitogen-activated protein kinase; MDA, malondialdehyde; MDC1, mediator of DNA damage checkpoint 1; MGMT, O6-methylguanine-DNA methyltransferase; NF-κB, nuclear factor-κB; NPC, nasopharyngeal carcinoma cells; NSCLC, non-small cell lung cancer; P53, tumor protein 53; PC-3, prostate cancer cells; PECAM-1, platelet endothelial cell adhesion molecule-1; PKC, protein kinase C; PGE, prostaglandin E; PIAS3, protein inhibitor of activated STAT3; PKB, protein kinase B; Prox-1, prospero homeobox 1; ROS, reactive oxygen species; SCC, squamous cell lung carcinoma; SCID, severe combined immune deficiency; SOD, super oxide dismutase; STAT3, signal transducers and activators of transcription 3; TGF, transforming growth factor; TPA, 12-O-tetradecanoylphorbol-13-acetate; TP53, tumor protein 53; VEGF, vascular endothelial growth factor; XIAP, X-linked inhibitor of apoptosis.
